# Resilience to HPV vaccine safety scares in seven countries

**DOI:** 10.7189/jogh.16.04148

**Published:** 2026-05-29

**Authors:** Mallory K Ellingson, Milkie Vu, Dur-E-Nayab Waheed, Swati Saxena, Fernando Pio de la Hoz, Natalia Pasternak, Yvonne Morrissey, Sharon Hanley, Michelle Fiscus, Tsetsegsaikhan Batmunkh, Paulo Almeida, Noel T Brewer

**Affiliations:** 1 Gillings School of Global Public Health, University of North Carolina, Department of Health Behavior, Chapel Hill, North Carolina, USA; 2 Lineberger Comprehensive Cancer Center, University of North Carolina, Chapel Hill, North Carolina, USA; 3 Feinberg School of Medicine, and Robert H. Lurie Comprehensive Cancer Center, Northwestern University, Department of Preventive Medicine, Chicago, Illinois, USA; 4 University of Antwerp, Centre for the Evaluation of Vaccination, Antwerp, Belgium; 5 American Cancer Society, New Dehli, India; 6 Universidad Nacional de Colombia, Bogota, Colombia; 7 Columbia University, Center of Science and Society, New York City, New York, USA; 8 HSE National Immunisation Office, Dublin, Ireland; 9 University of Aberdeen, Institute of Applied Health Sciences, Aberdeen, Scotland; 10 Association of Immunization Managers, Rockville, Maryland, USA; 11 National Cancer Council of Mongolia, Ulaanbataar, Mongolia; 12 Instituto Questao de Ciencia, Sao Paulo, Brazil

## Abstract

**Background:**

Resilience is the ability of a vaccination programme to return to high coverage after a disrupting event, such as a safety scare that undermines vaccine provision. To identify insights that can help countries achieve resilience in their vaccination programmes, we reviewed the experiences of countries that faced HPV vaccine safety scares.

**Methods:**

We developed case studies on HPV vaccine safety scares in three high-income (Denmark, Ireland, and Japan) and four middle-income countries (Brazil, Colombia, India, and Mongolia). The case studies included information on the safety scare, subsequent response, and impact on HPV vaccine uptake.

**Results:**

Reports of unfounded HPV vaccine safety concerns were amplified through traditional broadcast media coverage and perpetuated by parent groups and political leaders. After safety scares, five countries had widespread decreases in HPV vaccine coverage, one had regional drops in coverage, and two suspended introductions of the vaccine. Countries’ strategies varied, but typically involved coalitions of government entities, healthcare providers, community leaders, and cancer prevention organisations. All the countries were able to increase their HPV vaccine coverage or restart vaccine introduction.

**Conclusions:**

Safety scares were often imported from other countries, gained momentum through traditional media coverage, and were abetted by political leaders, delays by some vaccination programmes, and the absence of effective crisis communication plans. To build resilient HPV vaccination programmes, leaders should respond quickly to safety scares, develop a broad coalition (providers, political and community), share credible messages that emphasise cancer prevention, and communicate across traditional and social media.

National vaccination programmes can ideally achieve four things: high vaccine coverage, timely delivery, reduction in disease, and resilience to shocks. However, little is known about resilience, defined as the ability of vaccine programmes to return to higher coverage after destabilising events, such as supply shortages, wars, natural disasters, or safety scares [1-4]. Our paper focuses on safety scares for human papillomavirus (HPV) vaccine, given its importance to efforts to nearly eliminate cervical cancer [5]. Hundreds of studies have shown HPV vaccine is safe and effective in preventing cervical cancer and other HPV-related cancers [6–8].

Most countries have been successful in implementing HPV vaccination programmes. The rollout in Nepal in 2025 was a particular success, yielding 94% coverage for HPV vaccine first dose within the first 15 days in the focal population of 1.6 million girls [9,10]. Even some countries that have faced HPV vaccine safety scares, such as England, have been able to continue their vaccine programmes as planned [11]. However, others that faced safety scares related to HPV vaccine have experienced substantial destabilisation in their vaccination programmes [12–14]. Impacts have included decreases in coverage, suspension of vaccination programmes, missed opportunities to prevent HPV cancer deaths, and expensive and lengthy efforts toward recovery [12,14,15].

Though HPV vaccine safety scares are relatively uncommon, several have happened during HPV vaccine introduction or soon after. Rapid adoption of the vaccine globally is increasing opportunities for such scares. In the past 3 years, 33 countries have introduced HPV vaccine [16]. Another 45 countries will need to add HPV vaccine to their national vaccination programmes in the coming years. We sought to identify insights that can help vaccination programmes achieve resilience against HPV vaccine safety scares.

## METHODS

**Figure 1:**
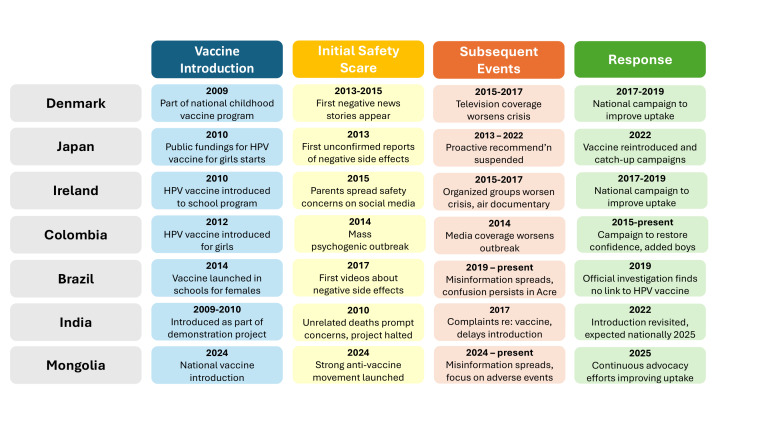
Summary of safety scare and response by country.

We developed case studies on HPV vaccine safety scares from experts on HPV vaccination programmes in seven countries (**Figure 1**). We first identified all countries that had experienced an HPV vaccine-related safety scare that lead to a disruption in coverage and reached out to experts in each country, where a safety scare is understood as any event where illness or death is attributed to HPV vaccine regardless of whether the illness or death is legitimately linked to HPV vaccine. Here, experts were understood as all individuals who, to some degree, have been involved in the HPV vaccination program in their respective countries.

We included three high-income (Denmark, Ireland, and Japan) and four middle-income countries (Brazil, Colombia, India, and Mongolia) where we could identify an expert on the local vaccine programme and collect adequate information on the safety scare and subsequent events. Three other have experienced a safety scare – Romania, Cote d’Ivoire, and the UK. We excluded the UK because they did not experience a disruption in coverage; for Romania and Cote d’Ivoire, we could not identify adequate documentation about the safety scare and its impact on HPV vaccine coverage.

Each expert received a standardised outline and wrote a case study on the HPV vaccine safety scare and recovery in their country. They provided information on each country’s HPV cancer burden and vaccination programme (country context), safety scare, subsequent response, and impact on HPV vaccine uptake (recovery). Study team members worked with these country experts to edit the case studies for length and clarity. Data on HPV vaccine coverage came from the World Health Organization’s (WHO) HPV Dashboard [16], supplemented by locally-provided regional data as needed, along with data on population size, cancer incidence, and vaccine programme characteristics [17–20] (Table S1 in the **Online Supplementary Document**). Study team members reviewed and discussed the case studies to identify common themes and insights for building resilient HPV vaccine programmes outside of policy changes alone (Table S2 in the **Online Supplementary Document**).

## RESULTS

### High-income countries

### Denmark

**Figure 2:**
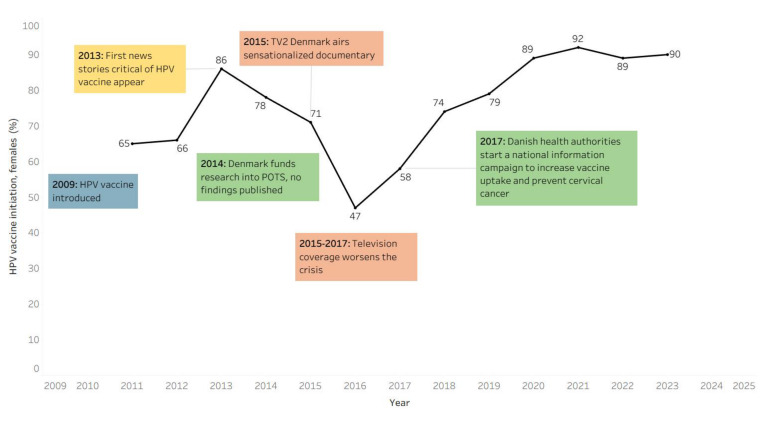
HPV vaccine coverage in Denmark.

Denmark introduced the HPV vaccine into its national childhood immunisation programme in 2009 (**Figure 1**). Initial HPV vaccine initiation rates were high, around 95% for eligible girls from 2009–2012 [14]. Starting in 2013, negative newspaper stories were associated with a decline in HPV vaccine uptake relative to before the stories appeared, and a negative television documentary and media coverage that followed it were associated with a 50% decline in one dose HPV vaccine coverage relative to baseline by 2017 (**Figure 2**) [21] and affected coverage of other vaccines [22]. A national information campaign coincided with recovery to pre-crisis levels of uptake as of 2025.

As of 2022, Denmark’s cervical cancer incidence is 9.7 per 100 000 women (**Table 1**) [17]. Cervical cancer is the 11th most prevalent cancer among Danish women, and the third most frequent cancer among women aged 15–44 years. Denmark recommends two doses of HPV vaccine for boys and girls ages 12–14 years. The vaccine is delivered by family physicians in primary care clinics at no cost to the family, while HPV vaccination coverage is tracked using a national registry.

**Table 1:** Country characteristics

	Denmark	Ireland	Japan	Colombia	Brazil	India	Mongolia
**Income**	HIC	HIC	HIC	UMIC	UMIC	LMIC	UMIC
**Population**	5.9 M	5.3 M	124.5 M	52.3 M	211.1 M	1.4 B	3.5 M
**Cervical cancer**							
Incidence per 100 000 population*	9.7	7.5	12.5	13.7	12.7	17.7	20.2
Deaths per 100 000 population	1.8	2.3	2.6	6.9	6.5	11.2	9.6
**Oropharyngeal**							
Incidence per 100 000 population*	4.1	1.6	0.9	0.9	2.0	1.6	0.5
Deaths per 100 000 population	1.1	0.8	0.3	0.3	1.2	1.0	0.5
**HPV vaccine**							
Year of introduction	2009	2010	2010, proactive recommendations suspended in 2013	2012	2014	Expected 2025	Pilot 2012, national 2024
Recommended age and doses	Boys and girls aged 12–14 years; two doses at least six months apart	Boys and girls aged 12–13 years since November 2022 a single dose of HPV vaccine is recommended for those aged 9–24 years	Girls aged 12–16 years; two-dose schedule for girls <15 years with HPV9 and three doses with HPV2 and HPV4	Girls and boys ages 9–17; two doses of the vaccine at least six months apart)	Boys and girls 9–14, 2014-24: two doses, six months apart; 2024 to present: single dose		Boys and girls aged 11 years, single dose schedule from 2024
Delivery setting	Healthcare centres	School or in a HSE clinic	Hospitals or medical clinics	The HPV-vaccine is administered in schools, hospitals, and health centres	Vaccination centres; in 2025, there were school vaccination campaigns	NA	Schools and primary care centres
Cost to patients	No cost for girls under the age of 18 years	No cost for patients, boys and girls	Free to girls in the target population	No cost for girls between 9 and 17 years of age	Free to target population ages 15–45 years can have access after evaluation for special circumstances in the *CRIEs*	NA	Free to target population ages 11-15 years

*CRIES* – *Centros de Referência para Imunobiológicos Especiais*, HIC – high-income country, HSE – Health Services Executive, LIC – low-income country, M – million, NA – not applicable, UMIC – upper-middle income country

*Cancer rates are age standardised per 100 000 persons as of 2022 from the International Agency for Research on Cancer [23].

From February 2013 to February 2015, stories critical of HPV vaccine appeared in Danish newspapers. Several articles suggested that HPV vaccine had serious side effects or suggested conflicts of interest for physicians who had advocated for HPV vaccination and its adoption into the Danish vaccine programme. From March 2015 to April 2017, television coverage paralleled a worsening of the crisis. In late March 2015, TV2 Denmark aired a sensationalised documentary that presented personal stories of young women who believed they had illnesses caused by HPV vaccination, and it suggested that Danish health authorities had not been forthcoming about the vaccine’s safety. Extensive media coverage followed on the possible association between HPV vaccine and severe adverse events. By the end of April 2015, Danish media outlets had referenced the TV documentary 170 times [24]. The documentary led to a rise in negative news articles from 140 negative news articles in 2014 to 1329 in 2015 [25]. According to a modelling study, the periods with negative media coverage left over 26 000 older girls unvaccinated who would have otherwise received the vaccine [24]. The missed doses may, over time, translate to as many as 180 avoidable cervical cancers and 45 deaths [14].

In 2016, the Danish Cancer Society, in collaboration with the HPV Prevention and Control Board, an international advisory group of experts on HPV, held a multi-stakeholder meeting to discuss the strategies to address the crisis [26]. From May 2017 to February 2019, Danish health authorities implemented a national information campaign ‘to prevent cases of cervical cancer in Denmark by increasing the uptake of HPV vaccination’ [14]. The campaign was a collaborative effort by the Danish Health Agency, the Danish Cancer Society, and the Danish Medical Association, funded by Health and Senior Citizen’s Committee of the Danish Parliament (**Table 2**). The campaign shared facts about HPV infections, cervical cancer, and HPV vaccination; used social media to disseminate information, primarily by creating a website and a Facebook page, while promoting Twitter hashtags, such as #stophpv; and made extensive use of personal stories from women who had suffered from cervical precancer or cancer. From the outset, the campaign website featured three personal stories and shared many additional personal stories on its Facebook page. Moreover, the campaign presented information that specifically targeted parents who had postponed HPV vaccination of their children. HPV vaccination appears to have made a full recovery for new age cohorts and those with low uptake (**Figure 2**).

**Table 2:** Characteristics of countries’ responses to safety scares*

	Denmark	Ireland	Japan	Colombia	Brazil	India	Mongolia
**Safety scare**							
Timing	2013–2015	2015–2016	2013–2022	2014–2015	2014	2010	2012; 2024–2025
Lowest HPV vaccine coverage†	2016: 47%	2016: 55%	2015: 0%	2016: 6%	2023: 42%‡	N/A	2024: 22%
**Response**							
Timing	2017–2019	2017–2019	2022 to present	2015 to present	2019	2022 to present	Ongoing
HPV vaccine coverage after response†	2024: 89%	2024: 73%	2024: 39%	2024: 60%	2024: 46%‡	N/A	April 2025: 37%§
Stakeholders involved	Danish Health Agency, Danish Cancer Society, Danish Medical Association	Human Papillomavirus Prevention and Control Board, Department of Health, Irish Cancer Society, Laura Brennan and the Brennan family	Ministry of Health, Labour and Welfare	Ministry of Health, HPV Prevention and Control Board, Colombia League Against Cancer	Ministry of Health, State Health Departments, Medical School – University of Sao Paulo	National Immunization Technical Advisory Group, Indian Medical Association, Federation of Obstetric and Gynaecological Societies of India	Ministry of Health, National Center for Communicable Diseases, National Cancer Council
Funding source	Health and Senior Citizens Committee, Danish Parliament	Department of Health	National Government		Federal and State governments		GAVI
Media and messaging used	Social media campaign focused on sharing personal stories of cervical cancer survivors	Public campaigns across digital and traditional channels including testimonies from vaccine advocates	Patient and caregiver information leaflets produced by Ministry of Health	Declarations from medical and scientific societies promoting HPV vaccine safety and effectiveness	None	School-located immunisation and community outreach programmes	Nationwide campaigns to promote HPV vaccination including posters and video content
Other responses		Fact sheets for providers and e-learning programmes, leaflets sent home to parents prior to child’s eligibility, consent obtained prior to vaccination	Fact sheets by vaccine manufacturers	Communication training for health care providers	After 2019, independent media outlets	Partnerships with medical societies to enhance physician training	Government-sponsored targeted training for healthcare providers

*Blank cells indicate that the information was not applicable or not publicly available.

†HPV vaccine initiation among girls. HPV vaccine coverage information is from the WHO HPV Vaccine Dashboard unless otherwise noted.

‡Lowest coverage in the state of Acre, site of the vaccine safety scare. Data provided by local authorities.

§Most up to date coverage following new initiatives taken in 2025. Data provided by local authorities.

### Ireland

Ireland introduced HPV vaccine into its immunisation programme in 2010 as part of its school immunisation programme. For the first five years, one dose HPV vaccine coverage was above 85% [16]. However, in 2015, parent groups began to share concerns about HPV vaccine through social media and a television documentary, which was associated with a 10% relative drop in one dose HPV vaccine coverage in 2015 relative to baseline and then a 50% relative drop in 2016 [16]. An organised campaign with extensive stakeholder engagement was associated with a recovery in one dose HPV vaccine coverage to above 70% [16].

Cervical cancer incidence in Ireland is 7.5 per 100 000 women (**Table 1**) [17]. Cervical cancer is the most frequently diagnosed HPV cancer (41%), followed by oropharyngeal cancer (31%). The HPV vaccine is provided to students for free in schools when they are in first year (age 12–13 years). A national immunisation registry (School Immunisation System) is used to record vaccines given.

The drop in HPV vaccine uptake in Ireland coincided with activities of parent groups that started in 2015 [27]. These groups made claims largely on social media that HPV vaccine caused serious long term side effects. At the time, the health service did not use social media as a form of communication, which allowed this misinformation to spread unhindered. Additionally, these groups worked with one of the national television stations to make a documentary (‘Cervical cancer vaccine – is it safe?’), where they shared patient stories of people claiming they were harmed by the vaccine, and broadcast it in December 2015 [27]. Shortly after, many parents withdrew from the HPV vaccination programme. One of the main parent groups spoke at conferences held by similar groups in Japan and Denmark and invited those parent groups to speak at conferences in Ireland. One of the parent groups continues to operate, but with a diminished reach. Most reported side effects were related to fainting and resolved quickly. Ireland monitored reported side effects for the vaccine and shared the data with the European Medical Agency, which ‘found no evidence that the overall rates of these syndromes in vaccinated individuals were different from expected rates’ [28].

In July 2017, the Irish Cancer Society launched the HPV Vaccination Alliance, a coalition of three dozen health and welfare organisations [29]. In November 2017, the HPV Prevention and Control Board held a meeting, on invitation from the government, to consider options to combat the widespread misinformation campaign [30]. A stakeholder engagement exercise found that parents needed time to research the vaccine. As a result, schools now send parents a leaflet each April to notify them their child will be eligible for vaccine starting in September. School teams also delayed the immunisation programmes until September or October to give parents more opportunities to research and speak to trusted health professionals. Because the exercise identified healthcare professionals and science teachers as trusted sources of vaccine information, fact sheets were developed containing updated information on the safety and effectiveness of HPV vaccine to be shared with these professionals. Public campaigns across digital and traditional channels ran from 2017 to 2019 supported by the coalition, the Minister for Health, and senior politicians. Vaccine advocates like Laura Brennan shared their personal experiences with cervical cancer; her death in 2019 made international news [31]. Since the implementation of these policies and campaigns, uptake of HPV vaccine has largely recovered (**Table 2**).

### Japan

Japan began funding HPV vaccine in 2010, achieving 70–80% coverage for girls ages 12–16 years [32]. It added the vaccine to their national immunisation programme in 2013, but proactive recommendations were suspended later that year due to reports of adverse events later investigated and not causally linked to HPV vaccine. The HPV vaccine coverage plummeted to less than 1% within a year [32]. Despite evidence supporting vaccine safety, proactive recommendations did not resume until 2022 and vaccination rates are still recovering. One-dose coverage for eligible females is estimated to be 39% as of 2024 (**Table 2**) [16].

Japan’s cervical cancer incidence and mortality are 12.5 and 2.6 per 100 000 women, respectively (**Table 1**) [17]. Until 2022, routine vaccination was optional for girls ages 12–16 years with either the bivalent or quadrivalent HPV vaccine and a three-dose schedule. The nine-valent HPV vaccine was introduced in 2023 with a two-dose schedule recommended for girls ages 12–14 years. The vaccine is delivered in medical clinics. Japan has no national vaccine registry.

In March 2013, one of Japan’s leading newspapers, *Asahi Shimbun*, published a story about a teen girl who had difficulty walking and doing math after HPV vaccination [33]. The story led other newspapers’ coverage of the vaccine to become markedly negative, and the coverage of the girls who reported complex regional pain syndrome became related to the vaccine [34]. Later in March 2013, the Japan Cervical Cancer Vaccine Victim Liaison Committee formed; backed by politicians, doctors, lawyers and parents, this group highlighted cases of girls experiencing widespread pain after vaccination, uncontrollable body movements, and school absences. Videos of affected girls spread through national media and social media, further fuelling public concern [35]. In April 2013, Japan added HPV vaccine to their national immunisation programme. In May 2013, Japan’s Vaccine Adverse Reactions Review Committee (VARRC) examined these cases and found no safety signal. However, the Victim Liaison Committee rejected the findings, increasing media coverage of alleged victims. In June 2013, just after the WHO’s Global Advisory Committee on Vaccine Safety affirmed HPV vaccine safety, VARRC suspended the proactive recommendation. The drop in HPV vaccine coverage was estimated in modelling studies to result in 5,000 additional cervical cancer deaths [36].

In December 2013, Japan came close to reinstating their proactive recommendation when VARRC classified reported symptoms as a ‘functional somatic syndrome’ unrelated to HPV vaccine. However, intense media backlash, with headlines like ‘Committee Says It’s All in Their Head’, and pressure from the Victim Liaison Committee stalled any action. This cycle repeated for years; VARRC findings dismissing a vaccine link were countered by media-driven public pressure, preventing the reinstatement of HPV vaccine recommendations until 2022.

In April 2022, Japan’s Ministry of Health, Labour, and Welfare resumed proactive HPV vaccination for girls ages 12–16 years. After the resumption of proactive recommendations, a three-year long catch-up scheme began for girls born between 1997 and 2007. HPV vaccine coverage has since recovered but only to about half its initial level [16].

### Middle-income countries

### Colombia

**Figure 3:**
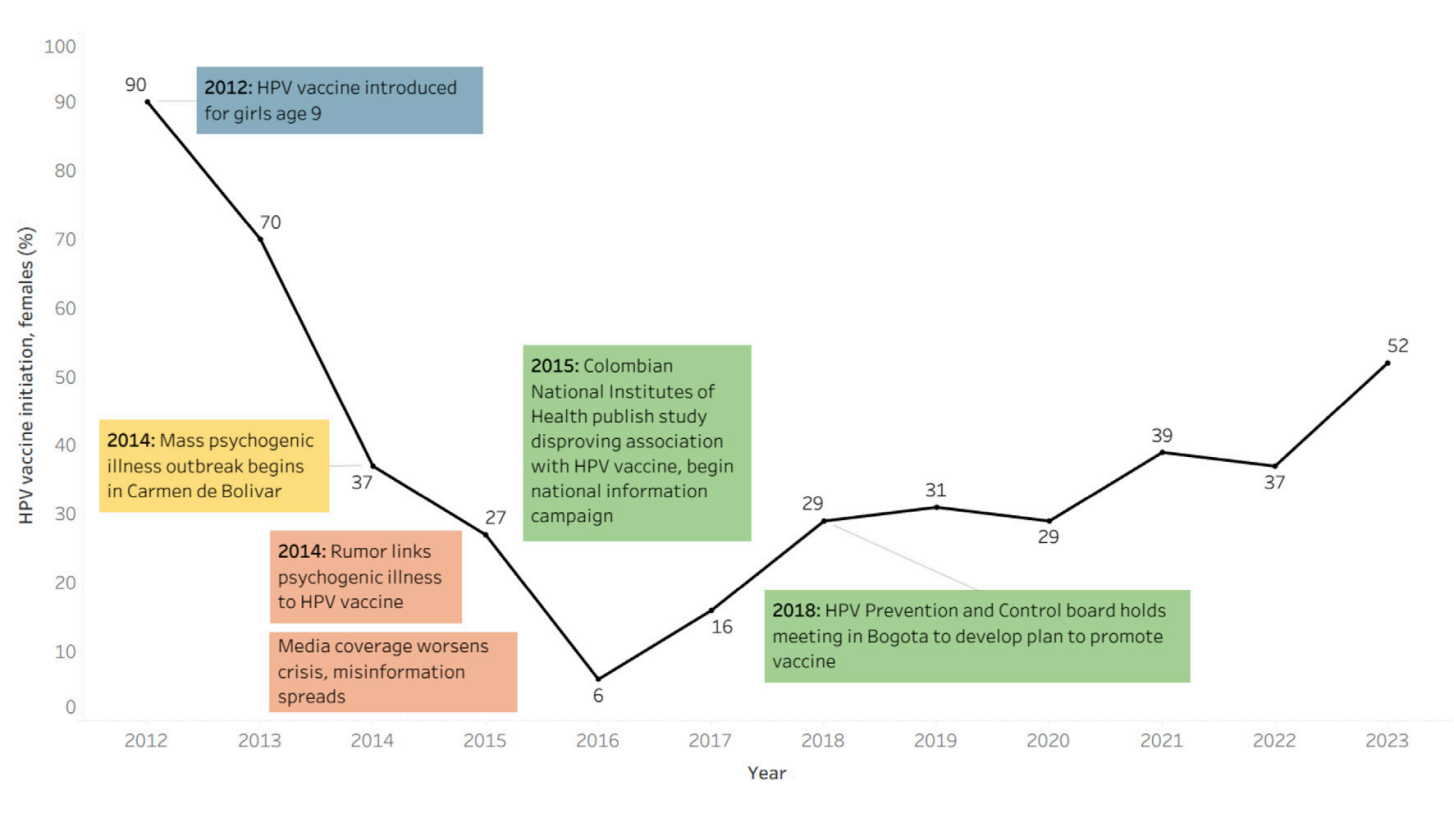
HPV vaccine coverage in Colombia.

Colombia introduced HPV vaccine first for girls aged nine years in 2012 and then extended the recommendation to girls aged 9–17 years to accelerate the control of HPV cancers and other diseases. By early 2014, HPV vaccination coverage was above 90% and more than 2.4 million girls had received at least one dose of the vaccine [37]. In mid- 2014, a widely publicised school-based incident of psychological illness prompted nationwide concern about HPV vaccine. One-dose coverage of the vaccine subsequently dropped to less than 20% [16]. Stakeholders met in 2018 to develop and implement a recovery plan. Coverage with one dose of HPV vaccine reached 60% as of 2024 (**Figure 3**) [16].

Colombia's cervical cancer incidence is 13.7 per 100 000 women (**Table 1**) [17]. Cervical cancer is the second most frequently reported cancer in women in Colombia and the fifth leading cause of death among women [38]. The country introduced HPV vaccination through school-located mass vaccination campaigns. Vaccine delivery continues primarily in schools, but it is also available at health centres and hospitals. As of 2023, HPV vaccine is free to children aged 9–17 years.

In May 2014, an episode of mass psychogenic illness occurred in Carmen de Bolivar (a small town on the Caribbean Coast) which affected more than 500 girls [39]. It started in January 2014 with one girl from a specific public-school reporting that she felt shortness of breath and fainted repeatedly, especially while attending school. Other girls slowly began to report the same clinical symptoms and rumours began to suggest the girls’ symptoms were due to HPV vaccination. The rumour may have originated with a naturalist physician consulted by parents unsatisfied with the local health system’s response. The crisis received extensive national radio, television, and newspaper coverage [39]. The number of girls affected grew as reporters, politicians, and health officials visited the town, peaking in August and September 2014. The events preceded one dose HPV vaccine coverage among eligible females dropping from 98% to 14% [37,39]. The absence of a structured communication plan, coupled with limited capacity to engage stakeholder groups, resulted in inadequate dissemination of information on HPV cancers during the crisis. This communication gap contributed to insufficient awareness and education among health workers, the media, and the public.

In February 2015, the Colombian *Instituto Nacional de Salud* released a report concluding that the symptoms were psychological in origin [40]. As national interest decreased, the crisis faded. However, the consequences have been long-lasting. In response, the government set in motion a plan to restore public confidence in HPV vaccines. The plan included declarations from relevant scientific and professional societies reassuring that HPV vaccines were safe and effective. An online course on vaccine safety by the Catalan Institute of Oncology and the Colombian Cancer Institute reached over 5000 healthcare workers. The response also included collaborations between parent associations, scientific societies, and non-governmental organisations interested in cancer control to find common ground to prevent and control cervical cancer. In June 2018, the Colombian League Against Cancer launched the Arauca Project, targeting one of the most affected regions with an integrated communication strategy that improved vaccination rates. In November 2018, the HPV Prevention and Control Board held a meeting in Bogota, at the invitation of the Ministry of Health, to develop strategies to fight vaccine hesitancy, and to promote HPV vaccination and screening [13,41]. These efforts helped to raise HPV vaccine coverage to roughly two third its initial level.

### Brazil

Brazil introduced the three-dose quadrivalent HPV vaccine in 2014 for girls aged 11–13 years, expanding down to those aged nine years in 2015. A two-dose regimen began in 2016. First dose coverage among females was 77% in 2015, but later declined, possibly due to misinformation, professional resistance, and anti-science rhetoric [16]. Reports of side effects in São Paulo and Acre led to further drops. Coverage remains high in São Paulo, but not in Acre (48%), where hesitancy persists despite investigations disproving vaccine safety rumours [42,43].

The incidence of cervical cancer in Brazil is 15.3 per 100 000 women [17]. It is the third most frequent cancer among women in Brazil overall and the sixth most frequent cancer overall excluding non-melanoma skin cancers [44]. One-dose quadrivalent HPV vaccine for aged 9–14 years is free through *Sistema Único de Saúde*, an integrated healthcare system, with coverage tracked through a national vaccine registry.

In 2014, eleven adolescent girls in Bertioga, São Paulo, reported headaches, fainting, and leg paralysis after HPV vaccination [45]. Neurologists and psychiatrists promptly investigated the reactions and found no link between vaccine and symptoms, classifying them as psychogenic reactions [45]. All individuals recovered promptly. National media initially sensationalised safety concerns, but later emphasised vaccine safety and the isolated nature of cases. Long-term HPV vaccine coverage in São Paulo remained unaffected, and by 2023, the state’s coverage exceeded the rate for the nation overall [43].

Also in 2014, in Rio Branco, Acre, dozens of adolescent girls reported headaches, leg pains, seizures, and fainting, often after viewing social media videos of similar events. No state or federal adverse events reports were filed until 2018 when the first serious events entered the surveillance system [46]. A mother’s petition prompted a public prosecutor’s inquiry, and in 2019, the Ministry of Health tasked the Institute of Psychiatry at *Hospital das Clínicas*, São Paulo, with investigating the incidents. The Institute attributed the cases to mass psychogenic illness [47]. Despite these findings, disinformation persisted, with some physicians alleging heavy metal contamination in HPV vaccines [48]. The stigma of psychogenic diagnoses, particularly in religious and low-income communities, fuelled distrust and hesitancy in the region [47].

HPV vaccine coverage dropped in the State of Acre from 100% in 2015 to 42% in 2023 [42]. Apart from investigating the case in 2019, the Ministry of Health took no specific action against the effects of the HPV vaccine safety scare. It adopted no specific communication plan, nor a global strategy besides the usual public vaccination calendar guidelines. The incident remained contained to the State of Acre, however, the consequences persist. A recent news investigation founds that, as of 2023, Acre health workers were still confused about HPV vaccine safety, and some preferred not to recommend it to patients at vaccination centres [49]. The Acre State government opened a special department in a public medical facility to care for teens diagnosed with psychogenic reactions. The staff included neuro-paediatricians, psychologists, psychiatrists, and gynaecologists [47]. Most of the adolescents rejected treatment, and the mothers did not accept the diagnosis. The psychiatrist’s report advised on the importance of providing training for local doctors and vaccination centres staff about psychogenic reactions to vaccination, but as of 2025 the service had not been created. Ministry of Health reports and national newspapers publicly clarified that the psychogenic reactions were isolated in nature, reaffirming the vaccine’s strong safety profile. Coverage remains low in the State of Acre, at just over half the rest of the country [43].

### India

India first introduced HPV vaccine in a demonstration project in 2009–2010. A safety scare during the project led the country to not recommend the vaccine for general use, so nationwide roll out is now expected by the end of 2025 [50], coinciding with the introduction of a domestically manufactured HPV vaccine. As of 2024, less than 1% of girls are vaccinated with one dose of the HPV vaccine [16].

India’s incidence of cervical cancer is 17.7 per 100 000 women, the second highest of the countries in our case studies (**Table 1**) [17]. It remains the second leading cause of cancer deaths among women in India. Overall, 23% of the world’s cervical cancer deaths occur in India. The HPV vaccine was not part of the routine vaccination schedule at the time of the safety scare. India is implementing an electronic vaccination registry nationally [51].

From 2009 to 2010, PATH and the Indian Council of Medical Research led HPV vaccine demonstration projects in Andhra Pradesh and Gujarat, states on the east and west coasts of India. The deaths of four girls enrolled in the project generated substantial public attention. In October 2009, a memorandum to the government by citizen advocates, academics, and experts raised concerns about the safety, efficacy, and cost effectiveness of HPV vaccine and called for a halt to all vaccine demonstration projects [12]. The project was suspended in April 2010, the same day a second memorandum was received. In June 2011, news coverage alleged that the deaths of the young tribal girls were caused by the vaccine. Heightened scrutiny of the demonstration project raised concerns about conflicts of interest, violation of moral standards, and unethical nature of recruitment and informed consent. A committee of three scientists from the All India Institute of Medical Sciences later found the deaths were due to poisoning, drowning, and fever, all unrelated to the vaccine. After the initial setback in 2009–2011, HPV vaccination became a low priority in India. It remained available in the private sector as an optional vaccine and was later introduced in a limited way for the states of Delhi in 2016 [52], Punjab in 2016 (in two districts) [53], and Sikkim in 2018 [54]. The vaccine again became controversial in 2017 when an affiliate of the Rashtriya *Swayamsevak Sangh*, a volunteer paramilitary organisation, called on *Swadeshi Jagran Manch*, a national political party, to file a complaint about alleged side effects of HPV vaccine. The complaint called the vaccine’s introduction an attempt to ‘pervert science’ and ‘sell the country to vested interests’ [55].

In September 2022, the Serum Institute of India launched Cervavac [56]. In December 2022, the National Technical Advisory Group on Immunisations recommended a countrywide introduction for girls aged 9–14 years, mainly through school-located immunisation and community outreach programmes. The recommendation included generating awareness through schools and supporting health teams for vaccination campaigns.

In January 2023, the Indian government instructed seven state governments to start preparations for the roll out of HPV vaccine for girls aged 9–14 years. Countrywide roll out is expected by the end of the year 2025 with a target of vaccinating 68 million girls [50]. Additional stakeholders have also joined efforts to promote HPV vaccine. The Indian Medical Association partnered with the Federation of Obstetric and Gynaecological Societies of India to encourage widespread HPV vaccination and enhance physician training. In February 2026, India launched a national HPV vaccine programme for girls aged 14 years. The vaccine is free of cost and delivered through clinics with a medical officer and internet connectivity to allow access to the national immunisation registry. A 90-day promotional campaign accompanied the launch [57].

### Mongolia

Mongolia first introduced quadrivalent HPV vaccine as a pilot programme in 2012. However, the vaccination campaign was halted the same year due to widespread misinformation attempting to link HPV vaccination to infertility, concerns about clinical trials, and resistance from some healthcare workers. In 2023, the national immunisation programme adopted a single dose, gender neutral recommendation for HPV vaccine for children age 11 years with the official nationwide rollout beginning in 2024. The programme again encountered significant opposition and public resistance, fueled by misinformation about vaccine safety. One dose HPV vaccine coverage among females was at 25% as of 2024 [16].

The incidence of cervical cancer in Mongolia is 20.2 cases per 100 000 women, the highest of our case studies and one of the highest in Asia [17]. It is the second most prevalent cancer among Mongolian women and the most frequent cancer among women aged 15–44 years. HPV vaccination is recommended for girls ages 11–15 years. Since 2024, the government has provided HPV vaccine free of charge through schools and primary health centres. Online vaccination records are maintained for eligible children.

The first pilot HPV vaccination programme in Mongolia began in March 2012. A three-dose schedule was administered to 9125 girls aged 11–15 years in two provinces and two districts, achieving 65% for three-dose coverage [58]. However, misinformation started to circulate in newspapers and on web sites almost immediately after the programme’s launch. The communication crisis was fuelled by messages claiming that the vaccine did not match the local disease aetiology, was untested in a clinical trial and thus had unknown efficacy, was delivered to use girls as guinea pigs by foreigners and was intended to sterilise Mongolian girls [59]. A prominent oncologist publicly advised parents not to vaccinate their children. In September 2012, the European branch of the nongovernmental organisation *Gal undesten holboo* held a press conference alleging that HPV vaccine caused infertility and had been rejected by Europe and other high-income countries. This wave of misinformation caused significant public confusion, resulting in the government suspending the HPV pilot vaccination programme later that month.

HPV vaccine studies conducted by the National Cancer Council of Mongolia in response to this wave of misinformation demonstrated high effectiveness, strong protection, and no negative impact on fertility. This locally generated evidence played a crucial role in informing the policy decision to re-introduce HPV vaccine. Based on the recommendations of the National Immunization Technical Advisory Group, Mongolia added HPV vaccine to its national immunisation schedule in December 2023.

In 2024, the government launched targeted training programmes for doctors, other healthcare providers, and schoolteachers in preparation for the national rollout. Comprehensive materials were developed (including training manuals, posters, and video content) and nationwide campaigns were organised to promote HPV vaccination. In November 2024, the nationwide rollout of HPV vaccine began again. In mid-November 2024, a vocal anti-vaccination movement re-emerged, whose activities included circulating a video of a woman falsely claiming that HPV vaccine contained toxic substances from aborted foetal tissues and false stories of adverse events associated with the vaccine. Initial coverage with one dose reached only 22%. A review of the lessons learned, and continuous advocacy efforts initiated from February 2025 have contributed to a steady increase in vaccination uptake, reaching about a third of eligible children.

## DISCUSSION

Our findings suggest that HPV vaccine safety scares are first and foremost social phenomena triggered by amplification of personal stories of harm, irrespective of whether they have a factual basis. In all seven safety scares, subsequent investigations found that the medical concerns were unrelated to HPV vaccine safety. While the details of safety scares are always specific to the local culture, politics, and healthcare context, several factors appear to help safety scares spread. First, they are often imported from other countries; stories from high-income countries played a powerful role when disseminated in middle-income countries [33]. Second, scares gain credibility and momentum when traditional media outlets carry parent stories to national or regional audiences. Third, the voices of political leaders and actions (or inaction) by vaccine programme leaders are consequential. Politicians and community leaders can sow doubt effectively, and vaccine programme leaders not engaging with a safety scare can allow public concerns to grow unchecked. Finally, not having an adequately resourced crisis communication plan and team limits programmes’ ability to address various scenarios before they become widespread [60,61]. Effective tools for such plans include prebunking of misinformation and rumours [62,63].

Once vaccine safety scares take root, programmes can still take action in addition to policy changes. We offer four insights from our case studies.

### Insight 1: respond within days not years

A delayed response can worsen the impact of a vaccine safety scare by ‘ceding the microphone’ to people spreading misinformation and alarm. Monitor misinformation on social media and be prepared to respond quickly. Being a regular part of the public discussion with credible information builds trust with the public, an important asset during a safety scare. Having a communication plan in place can aid in rapidly responding to a scare, as full communication campaigns take time to develop and implement, and funding for them is often delayed. The WHO Strategic Communications Framework offers support for developing a plan [66].

### Insight 2: develop a coalition of stakeholders from politics, health, and local communities

Many countries made effective use of coalitions of political leaders, healthcare providers, and their organisations, as well as cancer prevention organisations, cancer survivors, parents, and community and religious leaders. Engaging a multisector coalition can help reach multiple target audiences and engender public trust.

### Insight 3: share credible messages that emphasise cancer prevention

The most effective message topic is cancer prevention (*e.g.* ‘HPV vaccine prevents six cancers’) [65]. Offering training for healthcare providers on how to communicate effectively about HPV vaccine safety, effectiveness and cancer prevention can be highly effective [66,67]. The WHO has resources for developing and disseminating effective public messages about vaccines and safety [68]. Providing safety and effectiveness data can build trust; local data may be especially useful but can also be time consuming to produce, lessening its value.

### Insight 4: communicate across many media types

Vaccine programme leaders should combat misinformation both through traditional and social media. Successful campaigns in these case studies used a combination of social media messages and news appearances by cancer survivors among other approaches. We encourage interested programmes to also consult other vaccine resilience resources including a checklist and the Immunization Program Resilience Framework [2,3,69–71].

### Strengths and limitations

The strengths of our analysis include reliance on diverse experiences across country income and geographic regions as well as substantial data on vaccine uptake impact and programmatic details. Limitations include the absence of case studies for Africa or North America or from low-income countries and selective sampling approach informed by which countries have experienced safety scares. The actions identified above are likely still applicable in these settings; however, further work is needed to adapt these responses to an HPV vaccine safety scare to the specific context. Dynamics related to vaccine confidence and social media sharing of misinformation may have changed since these countries faced their safety scares, including during the COVID-19 pandemic. Finally, our brief case studies necessarily elide some of the rich details of the safety scares and recovery and are reliant on expert recollection.

## CONCLUSIONS

A timely and clear response from leaders can help build resilience in response to safety scares. Doing so requires collaboration among policymakers, local health organisations, and other key stakeholders to create a comprehensive campaign that can combat misinformation across multiple forms of media and emphasises cancer prevention. It also requires the engagement of trusted messengers such as providers, teachers, clergy or other community leaders. Through these efforts, it is possible to achieve high HPV vaccine coverage after a safety scare. The remnants of these safety scares can linger and may pose an ongoing threat of reactivation within and across countries. Therefore, even in countries that have previously experienced a safety scare it is important to be prepared.

## Additional material


Online Supplementary Document
