# Supplementary material for: Resilience to HPV vaccine safety scares in seven countries
Source: J Glob Health. 2026 May 29;16:04148. doi: 10.7189/jogh.16.04148 (PMC13221712; doi:10.7189/jogh.16.04148)
Supplement: Online Supplementary Document [file jogh-16-04148-s001.pdf]

Supplement to: Cite as: Ellingson MK, Vu M, Waheed N, Saxena S, de la Hoz FP, Pasternak N, Morrissey Y, Hanley S, Fiscus M, Batmunkh T, Almeida P, Brewer NT. Resilience to HPV vaccine safety scares in seven countries. J Glob Health. 2026;16:04148.

Table S1. Data sources for all vaccine coverage estimates

Country	Estimate	Specific Measure	Source
**Denmark**	*"Initial HPV vaccine initiation rates were high, around 95% for eligible girls from 2009-2012."*	HPV vaccine initiation was defined as the total number of initiations in a month divided by the number of girls who became eligible for HPV vaccination in the same calendar year, then multiplied by twelve to annualize the rates	Danish Ministry of Health via the Hansen et al. *Vaccine* (2019)[12]
	"… 50% decline in HPV vaccine uptake relative to baseline by 2017."	HPV vaccine one dose coverage among females by age 15	WHO HPV Dashboard[14]
**Ireland**	*"For the first five years, HPV vaccine uptake was above 85%."*	HPV vaccine one dose coverage among females by age 15	WHO HPV Dashboard[14]
	"… which was associated with a 10% relative drop in uptake in 2015 relative to baseline and then a 50% relative drop in 2016."	HPV vaccine one dose coverage among females by age 15	WHO HPV Dashboard[14]
**Japan**	*"… achieving 70-80% coverage for girls ages 12-16 years."*	One dose uptake rates by eligible birth cohort for girls ages 12-16 years	Deaprtment of Infection Control, Sapporo Health Board via Hanley et al. *Lancet* (2015)[29]
	"HPV vaccine coverage plummeted to less than 1% within a year"	See above.	See above.

	"One-dose coverage across the whole female population is estimated to be 39% as of 2024."	HPV vaccine one dose coverage among females by age 15	WHO HPV Dashboard[14]
**Colombia**	*"By early 2014 HPV vaccination coverage was above 90%..."*	One dose coverage among girls ages 9-17 in 2013	Colombian Health Ministry[34]
	"One-dose coverage of the vaccine quickly dropped to less than 20%	HPV vaccine one dose coverage among females by age 15	WHO HPV Dashboard[14]
	"…HPV vaccine initiation dropping from 98% to 14%"	One dose HPV vaccine coverage among eligible females	Colombian Health Ministry via Simas et al. *Hum Vaccine Immuno* (2019)[36]
	"Coverage with one dose of HPV vaccine reached 60% as of 2024."	HPV vaccine one dose coverage among females by age 15	WHO HPV Dashboard[14]
**Brazil**	*"First dose coverage among females was 77% in 2015..."*	HPV vaccine one dose coverage among females by age 15	WHO HPV Dashboard[14]
	"HPV vaccine coverage dropped in the State of Acre from 100% in 2015 to 42% in 2023"	HPV vaccine one dose coverage among females by age 15	Instituo Ciencia de Questao[41]
	"Coverage remains high in Sao Paolo but not in Acre (48%)..."	HPV vaccine one dose coverage among females by age 15	Instituo Ciencia de Questao[41]
**India**	*"As of 2024, less than 1% of girls are vaccinated with one dose of the HPV vaccine."*	HPV vaccine one dose coverage among females by age 15	WHO HPV Dashboard[14]
**Mongolia**	*"One dose HPV vaccine coverage among females was at 25% as of 2024."*	HPV vaccine one dose coverage among females by age 15	WHO HPV Dashboard[14]

	"Initial coverage with one dose reached only 22%"	One dose among boys and girls age 11 years	Provided by country expert.

Table S2. Sources for themes and insights

Themes	Insights	Case Study	Sample Quotes from Case Studies
Safety scares are often imported from other countries	**Insight 3.** Share credible messages that emphasize cancer prevention<br><br>**Insight 4.** Communicate across many media types	Ireland	*"One of the main [anti-HPV vaccine] parents groups spoke at conferences held by similar groups in Japan and Denmark and invited those parent groups to speak at conferences held in Ireland"*
		Mongolia	*"In September, the European branch of the nongovernmental organization, "Gal undesten holboo" held a press conference allegine that HPV vaccine caused infertility and had been rejected by Europe and other high-income countries"*
		Japan	From Larson et al. *Hum Vaccines Immunother* (2014):[30]<br><br>*"The news of Japan's suspension of the HPV vaccine recommendation has traveled globally through online media and social media networks…"*
Scares gain credibility and momentum when traditional media outlets carry parent stories to national or regional audiences	**Insight 1.** Respond within days, not years<br><br>**Insight 4.** Communicate across many media types	Denmark	*"From February 2013 to February 2015, stories critical of HPV vaccine appeared in Danish newspapers. Several articles suggested that HPV vaccine had serious side effects…"*<br><br>*"From March 2015 to April 2017, television coverage worsened the crisis. In late March 2015, TV2 Denmark aired a sensationalized documentary.."*
		Ireland	*"Additionally, these [anti-HPV vaccine parent] groups worked with one of the national television stations to make a documentary (Cervical cancer vaccine – is it safe?) sharing patient stories of people claiming they were harmed by the vaccine and its broadcast in December 2015."*

		Colombia	*"The crisis received extensive national radio, television and newspaper coverage."*
The voices of political leaders and actions (or inaction) by vaccine program leaders are consequential	**Insight 1.** Respond within days, not years<br><br>**Insight 2.** Build a coalition of stakeholders from politics, health and local communities<br><br>**Insight 3.** Share credible messages that emphasize cancer prevention	India	*"The vaccine became controversial again when an affiliate of the Rashtriya Swayamsevak Sangh, a volunteer paramilitary organization, called on Swadesh Jagran Manch, a national political party, to file a complaint about the alleged side effects of HPV vaccine."*
		Brazil	*"Also in 2014, in Rio Branco, Acre, dozens of adolescent girls reported headaches, leg pains, seizures, and fainting, often after viewing social media videos of similar events. No state or federal adverse events reports were filed until 2018 when the first serious events entered the surveillance system"*
		Japan	*"Backed by politicians, doctors, lawyers and parents, [the Japan Cervical Cancer Vaccine Victim Liaison Committee] highlighted cases of girls experiencing widespread pain after vaccination, uncontrollable body movements, and school absences.*
Not having an adequately resourced crisis communication plan and team limits programs' ability to address various scenarios before they become widespread	**Insight 1.** Respond within days, not years<br><br>**Insight 4.** Communicate across many media types	Brazil	*"The Ministry [of Health] adopted no specific communication plan, nor a global strategy besides the usual public vaccination calendar guidelines."*<br><br>*"*
		Colombia	*"The absence of a structured communication plan, coupled with limited capacity to engage stakeholder groups, resulted in inadequate dissemination of information on HPV cancers during the crisis."*
		Ireland	*"At the time, the health service did not use social media as a form of communication, which allowed this misinformation to spread unhindered."*
